# Prospective randomized controlled trial: early weight bearing after conservative treatment of Weber B ankle fractures (pancake trial)

**DOI:** 10.1007/s00590-023-03651-6

**Published:** 2023-09-02

**Authors:** R. C. Stassen, S. Franssen, B. Meesters, B. Boonen, E. R. de Loos, R. van Vugt

**Affiliations:** 1 Department of Traumatology, Zuyderland Medical Centre, Henri Dunantstraat 5, 6419 PC Heerlen, The Netherlands; 2 Department of Orthopaedic Surgery, Zuyderland Medical Centre, Henri Dunantstraat 5, 6419 PC Heerlen, The Netherlands

**Keywords:** Lateral transsyndesmotic ankle fracture, Cast, Walking boot, Permissive weightbearing, Functionality

## Abstract

**Purpose:**

Different studies have shown that weightbearing is safe in stable transsyndesmotic, isolated lateral simple ankle fractures. Despite this evidence, AO guidelines still recommend immobilization with above-the-knee cast for 4–6 weeks for these fractures. The objective of this study was to compare the outcomes of mobilization and weightbearing to those of immobilization and non-weightbearing in patients with stable transsyndesmotic, lateral isolated simple ankle fractures.

**Methods:**

Fifty patients were randomly assigned to permissive weightbearing in a walking boot or non-weightbearing immobilization using a below-the-knee cast. Primary outcome was ankle functionality as scored by the Olerud-Molander Ankle Score (OMAS). Secondary outcomes were radiological displacement of fracture, range of motion (ROM), calf circumference, and RAND 36-item health survey. Patients were in follow-up for 24 months.

**Results:**

Ankle functionality after six and twelve weeks was significantly higher for the intervention group, with respectively 30 points (*p* = 0.001) and 10 points (*p* = 0.015) of difference. ROM improved significantly in the intervention group after six weeks. All fractures showed radiological progression of fracture healing. RAND 36-item showed differences in both physical (60.3 vs. 46.3, *p* = 0.017) and mental (78.5 vs. 58.2, *p* = 0.034) components in favor of the intervention group. In 16% of patients who initially showed stable fractures on radiographic imaging, joint dislocation was identified on weightbearing radiographs prior to randomization, leading to exclusion.

**Conclusion:**

Weightbearing and mobilization using a walking boot may be a safe treatment for patients with stable Weber B fractures.

**Supplementary Information:**

The online version contains supplementary material available at 10.1007/s00590-023-03651-6.

## Introduction

### Background and objectives

Ankle fractures are common traumatic injuries requiring adequate management. In the USA, approximately 673.214 ankle fractures occurred between 2012 and 2016, with an incidence of 4.22/10.000 person-years [1]. Isolated malleolar fractures account for approximately seventy percent of all ankle fractures, of which lateral fractures, also known as ‘Weber B’ fractures, are the most common [2]. In the assessment of ankle fractures, stability of the talocrural joint is crucial, as it dictates the indicated treatment [3–5]. Only when syndesmotic and associated deltoid ligament injuries are ruled out, conservative treatment including weightbearing can be considered in this type of fracture. Although many already agree on the conservative treatment of stable Weber B fractures (AO type 44B1.1 and 44B1.2 fractures), using orthoses, the Arbeitsgemeinschaft für Osteosynthesefragen (AO) guidelines still suggest immobilization in an above-the-knee cast for 4–6 weeks [6–8].

When patients are restricted to a prolonged period of immobilization, adverse effects regarding recovery, increased physical effort, and risk of thrombosis are observed [9, 10]. Over the years, non-weightbearing has been the treatment of choice, however biomechanical studies have shown mechanical stimulation of fracture fragments to improve fracture healing in stable fractures [11–13]. Previous clinical studies have demonstrated the safety and effectiveness of weightbearing in the conservative treatment of stable Weber B ankle fractures [13–15].

This study aims to compare mobilization and weightbearing using a walking boot to immobilization and non-weightbearing in a below-the-knee cast. Patients will be assessed based on functional, anatomical and health state outcomes.

## Patients and methods

### Trial design

This randomized controlled trial was conducted in a high volume trauma center in [location blinded]. Interventional treatment consisted of six weeks of mobilization and weightbearing using a Rebound® walker, while the control group was designated to six weeks of immobilization and non-weightbearing with a below-the-knee cast. The study was conducted according to the CONSORT guidelines [16]. Trial protocol and statistical analysis plan have been included in Online Appendix 1. This study was approved by the local medical ethical committee ([code not enclosed due to blinding reasons], date of approval: April 4, 2018) and registered [register not enclosed due to blinding reasons].

### Sample size calculation

We determined that a total of 46 patients would be required to provide a 80% power. With an effect size of 15 points of difference on the OMAS and a variance of 324 (square of SD in study by Dehghan et al.), 23 patients per group were required [17]. Sample size was calculated using ‘EpiTools epidemiological calculator’ (Epitools online, Ausvet, Canberra, Australia) by inserting means and standard errors of a study investigating the same outcome measure [17]. To correct for lost to follow-up, a 10% margin for error was added, bringing the total amount of inclusions to 50.

### Participants

From March 2018 to October 2019, patients aged ≥ 16 years old visiting the emergency department with an isolated Weber B fracture (Lauge Hansen supination-eversion stage 2-4A) were screened for eligibility. Patients were eligible for inclusion if non-weightbearing radiographs showed congruent ankle joint on mortise view (medial clear space < 6 mm and ≤ 1 mm wider than the superior clear space). Weightbearing radiographs (weightbearing and non-weightbearing) were acquired after one week. If the ankle was deemed stable after one week, informed consent was signed and patients were randomized. Patients showing a widened medial clear space (> 6 mm) on radiographs were excluded and underwent open reduction and internal fixation. Exclusion criteria were ankle injuries other than isolated Weber B fractures, bilateral fractures, delay in presentation of more than 14 days after injury, and contralateral amputation of upper leg, lower leg or foot.

### Randomization

After written consent was obtained, patients were randomly appointed to the control or intervention group (1:1 ratio) by the investigators (SF, RS) using an online randomization tool, provided by ‘Researchmanager’ [18]. For concealed allocation of the participants, a computer-generated list of random numbers was used, which was accessible by both investigators. Although blinding of patients and researchers is desirable, this was not deemed feasible due to the nature of the intervention.

### Interventions

A cast below the knee was applied by a trained plaster technician in the non-weightbearing group. The cast was applied from a few centimeters below the tuberositas tibiae to the base of the toes. The ankle was fixed in a plantigrade position. Patients assigned to cast group were specifically instructed not to bear weight on the affected limb. If any cast-related complaints occurred during treatment, a new cast was fitted. The weightbearing group was fitted a walking boot (Rebound® Air Walker, Össur, Reykjavik, Iceland). These patients were instructed on permissive weightbearing and use of the walking boot. Instructions included the removal of the walking boot when not weightbearing to mobilize the affected ankle.

Although recent studies suggest thromboprophylaxis is no longer indicated in permissive weightbearing [19], both groups were prescribed daily use of Dalteparin 5.000 IE for the duration of the intervention in order to minimalize the risk of bias. After six weeks, cast or walking boot were removed and patients were instructed to functionally use the ankle again. There was no transition from walking boot or cast to a brace, as patients were instructed permissive weightbearing. No limitations as to rehabilitation were imposed, and no formal physical therapy was prescribed for these patients.

### Outcome measures

Primary outcome measure was the Olerud-Molander Ankle Score (OMAS; ordinal scale 0–100 with intervals of 5 points, whereas lower scores indicate worse outcome) after six weeks to compare ankle functionality in both groups. The OMAS is a frequently used ankle functionality score based on patient-reported measures [17, 20–23]. OMAS was assessed at study start (week 0) and during follow-up (week 6 and week 12). At week 0, patients were asked to complete OMAS with regard to function before fracture to gain a baseline functionality. At 6-week follow-up, OMAS questionnaire was immediately given to patients after removal of cast or walking boot. As recent studies [13, 17] have shown a regression toward full recovery for both interventions over a period of 12 months, primary outcome was expected to be significantly different after six weeks. Secondary outcome measures were displacement of fracture after 6 weeks (based on radiographs; dichotomous variable yes/no), range of motion (ROM) of the ankle using a goniometer, calf circumference (as measured 10 cm’s below lower edge of the patella with a leg in extension) and short form 36-item (SF-36) health survey (version 1.0) as patient-reported outcome measure (PROM) to compare health-related quality of life. The SF-36 covers eight health domains, scores range from 0–100 with higher scores defining a favorable health state [24]. All secondary outcome measures were assessed at each follow-up moment except for radiological assessment (not done at week 12). Fracture displacement at six weeks was analyzed by both trauma or orthopedic surgeon and radiologist on call. Complications including cast compression, venous thromboembolism, hyperesthesia, and fracture malunion were recorded as adverse events.

### Follow-up

Physical follow-up was planned at week 0, and 6 and 12 weeks after trauma. After these visits, patients were instructed to contact the primary investigator in case of medical complaints. Patient files were checked both 12 months and 24 months after randomization. Patients were randomized in week 0. Radiological imaging evaluation was conducted in week 0 (weightbearing anteroposterior (AP) and non-weightbearing AP, lateral and mortise radiographs) and after six weeks (non-weightbearing AP, lateral and mortise radiographs). During every follow-up moment, physical examination (ROM, calf circumference) of the ankle was conducted, and patients were required to complete OMAS and RAND SF-36 questionnaire.

### Statistical methods

All statistical analysis were performed using SPSS (IBM Corp. IBM SPSS Statistics for MacOS, Version 25.0, Armonk, NY, USA) and were conducted according to the intention-to-treat principle. In order to prevent type I errors, per-protocol analysis was also conducted. Every outcome measure was compared between the control and intervention arms of the study. If there was equal variance of continuous variables, independent samples T test was used. Mann–Whitney U test was performed for the remainder of continuous variables. Wilcoxon ranked test was used for the analysis of non-normally distributed dependent variables. SF-36 questionnaire results were translated to percental values using the Orthotoolkit SF-36 software [25].

## Results

**Fig. 1:**
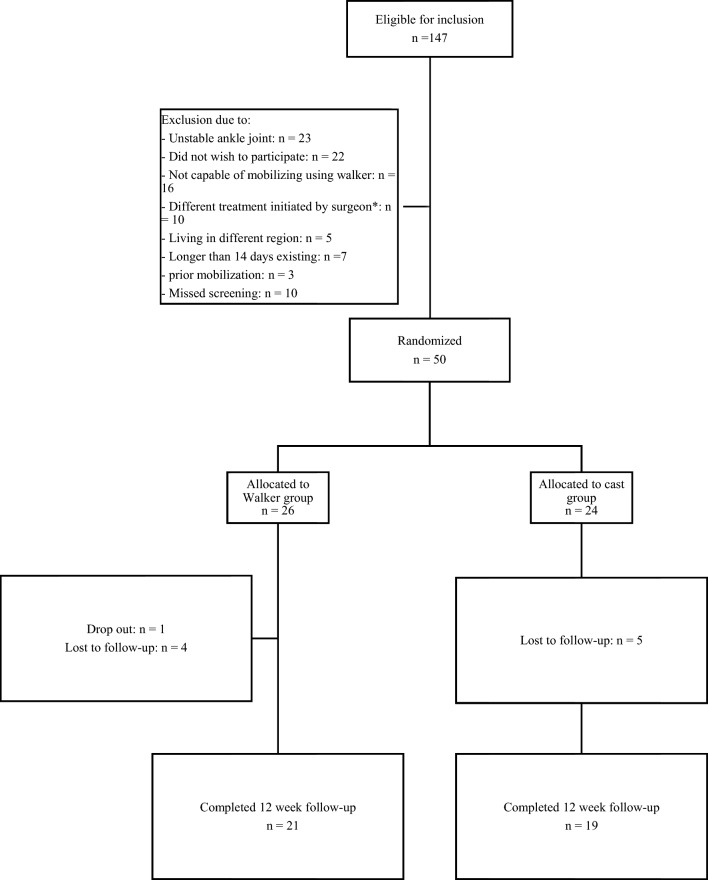
Flow diagram describing patient enrollment and randomization during this trial. *Different treatment initiated by surgeon: 3 weeks non-mobilization in a below-the-knee cast, followed by 3 weeks of mobilization using a below-the-knee cast

A total of 147 patients were eligible for inclusion. Fifty patients were randomized. Of these, 24 were randomized in the non-weightbearing group and 26 in the walking boot group. Weightbearing radiographs excluded 23 (15.6%) of 147 patients due to ankle joint instability. Twenty-two (14.9%) of 147 patients were not willing to participate. Other reasons for exclusion can be found in Fig. 1. Patient baseline characteristics are presented in Table 1. Forty patients completed the 12-week follow-up. Ten patients were lost to follow-up (no-show after multiple attempts of contact). Of those, five patients were allocated to the walking boot group. Protocol violations occurred in three patients. These patients mobilized without walking boot after three weeks. No action was taken as the violations happened in settings that could not be influenced. Three participants included in the cast group returned with the request of allowing weightbearing on the cast because of practical reasons (standing job) and the absence of pain (twice). A cast shoe was fitted for those patients for the remainder of two weeks. The last four patients were not able to fully attend the physical 12-week follow-up visit, due to the COVID-19 pandemic. Hospital policy demanded telephonic consultation in order to assess the need for in-hospital examination. As patients reported no complications, their final follow-up was waived. Questionnaires (OMAS & SF-36) were acquired by telephone. Within six months, four patients returned to the hospital due to joint stiffness. Of these patients, three were in the control group. Patient did not experience other complications within 24 months.

**Table 1:** Baseline characteristics

	Overall (*n* = 49)	Cast (*n* = 24)	Walker (*n* = 25)
*Sex*			
Male (*n* (%))	24 (48%)	7 (29.2%)	17 (68%)
Female (*n* (%)	25 (52%)	17 (70.8%)	8 (32%)
Age (mean (± SD))	52 (± 17)	51 (± 15)	52(± 19)
Smoker (*n* (%))	11 (22%)	5 (20.8%)	6 (23.1%)
Retired (*n* (%))	23 (47%)	9 (37.5%)	14 (53.8%)

### Ankle functionality

Baseline OMAS was comparable between intervention and control group (Table 2). A statistical significant difference was observed between study arms after 6 and 12 weeks, respectively 30 points (*p* < 0.001) and 10 points (*p* = 0.015) in favor of the intervention arm. These results were consistent with the a priori calculation. ROM at baseline (1 week after fracture) was similar between both groups. After 6 weeks, the intervention group showed a significant improvement of range of motion, whereas no difference was observed in the control group. After 12 weeks, this difference was no longer present in both study groups.

**Table 2:** Outcome measures of patients having completed follow-up

	Overall (*n* = 49)	Cast (*n* = 24)	Walker (*n *= 25)	*P* value
Olerud-Molander ankle score*				
Baseline (median (IQR))	100 (100–100)	100 (100–100)	100 (100–100)	
6 weeks (median (IQR))	55 (40–72)	40 (30–50)	70 (65–80)	*P* < 0.001
12 weeks (median (IQR))	80 (60–90)	75 (43–80)	85 (64–95)	*P* = 0.015
Range of motion*				
Baseline (median (IQR))	35 (26–42)	36 (27–42)	35 (26–43)	
6 weeks (median (IQR))	43 (30–50)	32 (28–44)	48 (42–52)	*P* < 0.001
12 weeks (median (IQR))	51 (46–56)	50 (46–53)	54 (48–57)	
Calf circumference**				
Baseline (mean (± SD))	37.0 (3.0)	36.3 (2.5)	37.6 (3.3)	*P* > 0.1
6 weeks (mean (± SD))	35.4 (3.2)	34.7 (2.8)	36.5 (3.5)	*P* > 0.1
12 weeks (mean (± SD))	36.6 (3.3)	36.6 (2.8)	36.7 (3.8)	*P* > 0.1
SF-36 Health outcome score*				
Mental component baseline (median (IQR))		91.9 (10.5)	88.9 (19.1)	*P* > 0.1
Physical component baseline (median (IQR))		86.6 (9.2)	90.9 (17.3)	*P* > 0.1
Mental component 6 weeks (median (IQR))		58.2 (32.1)	78.5 (19.2)	*P* = 0.034
Physical component 6 weeks (median (IQR))		46.3 (13.8)	60.3 (34.4)	*P* = 0.017
Mental component 12 weeks (median (IQR))		84.1 (21.2)	91.8 (22.3)	*P* > 0.1
Physical component 12 weeks (median (IQR))		63.8 (36.7)	75.9 (32.3)	*P* > 0.1

*Measured using Mann–Whitney* U* test

**Measured using independent samples* T* test

Calf circumference was normally distributed among groups. No baseline difference was seen between both groups. There was a statistical significant decrease in calf circumference after six weeks, both the intervention as control group in comparison with baseline measurement (Table 2). There was no difference between both groups after 12 weeks.

### Fracture healing

**Fig. 2:**
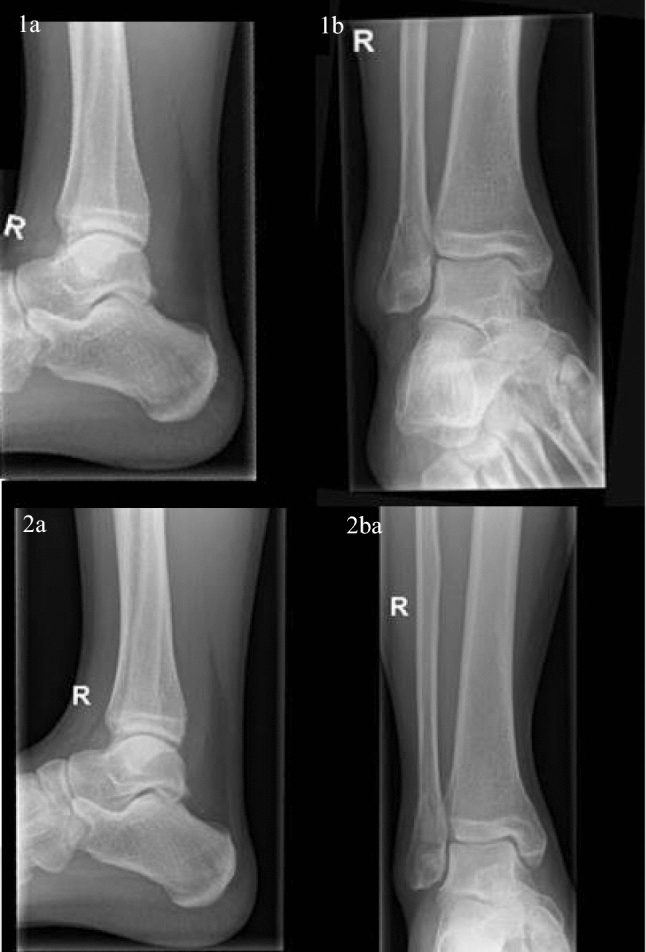
Radiographs taken posttraumatic and after six weeks of permissive weightbearing. Image 1a and b were obtained 2 days after trauma for this patients. Lateral view (image **1a**) of ankle shows no evident signs of fracturing. Mortise (image **1b**) view shows an evident fracture without widening of the medial clear space. Image **2a** & **b** were obtained 6 weeks after permissive weightbearing. Lateral view (image **2a**) shows no signs of fracture displacement. Mortise view (image **2b**) shows no signs of fracture displacement and show signs of callus bridging

Radiologic follow-up after six weeks showed no fracture displacement in any patients. All fractures showed signs of callus bridging of the fracture site after 6 weeks. For illustration: Figure 2 shows radiographs taken two days after trauma and after six weeks of permissive weightbearing. No delayed union was observed in any patient after 12 weeks. Five patients (12%) reported severe pain during treatment (Visual Analog Scale for pain > 7). Cast (*n* =  4) or walking boot ( *n* = 1) was removed between week 3 and 4 of the study, and control radiographs were obtained. In none of these cases fracture displacement was observed, and pain was regarded as pressure pain due to the cast. For the patient appointed to the intervention group, no obvious reason was found, and the patient requested change of therapy to cast. In these five patients, new casts were fitted, and all completed the 5-week initial therapy. The patient switching from walking boot to cast was analyzed according to intention-to-treat principle.

### SF-36

No statistical differences between both treatment groups were observed at baseline. There was however a statistical significant difference after six week in the intervention group compared to the control group (Table 2). This difference was observed in both the mental (78.5 vs 58.2,* p *= 0.034) and physical (60.3 vs 46.3, *p *= 0.017) domains. No differences were found between both groups after 12 weeks.

### Harms

No serious adverse events where observed. In five patients, unintended events were registered. Four events were related to contingency hospitalization due to pre-existent medical conditions. In one case, a patient randomized to the cast group developed posttraumatic complex regional pain syndrome. No delay of fracture union was observed in this patient. This patient was referred to our specialized pain clinic and treated with amitriptyline after which she reached a pain-free state approximately one month after completion of follow-up.

## Discussion

Permissive weightbearing using a walking boot resulted in both better ankle function and health-related quality of life after six weeks when compared to non-weightbearing immobilization using a below-the-knee cast.

This study is not the first to compare mobilization and weightbearing to immobilization and non-weightbearing [8, 13, 15, 17]. Most recent studies investigating alternative treatment options in stable type B ankle fractures compare functional bracing to casting, demonstrating functional bracing to be non-inferior to casting [13, 15]. Only few studies have described the use of a walking boot for this type of fracture. Brink et al. described the difference between functional bracing and walking boot for stable ankle fractures, whereas both treatments showed good fracture healing, although the walking boot provided better pain relief, increased range of motion, and earlier return to work[26]. Our study's results are consistent with those reported in multiple studies, including the research of Zeegers et al. in 1989, which demonstrated the safety of mobilizing Weber B fractures [27]. Despite the recent publications of evidence supporting the safety of early mobilization in patients with stable Weber B fractures, cast immobilization for a period six weeks remains the official treatment as indicated by the AO [7, 13, 15]. Financial reasons may be a possible explanation as to why the AO foundation still recommends a cast, as the price of cast is significantly lower than a walking boot or another form of orthosis. An alternative explanation could be that orthopedic and trauma surgeons prefer minimizing the risk of fracture displacement over the potential adverse effects related to prolonged immobilization, including skin irritation, joint stiffness, and thromboembolisms [28]. Although not actively investigated in this study, potential advantages of the walking boot consist of increased patient satisfaction, fewer problems with self-care, and reduced dependence on help services and faster return to work. These advantages will be of interest for future studies.

The comparison of calf circumference measurements between treatment groups did not yield any statistically significant differences at any of the follow-up intervals. It should be acknowledged that this study was not designed with sufficient statistical power to accurately determine the significance of any differences in calf circumference between groups. Therefore, it would be premature to draw definitive conclusions regarding the significance of any observed differences in calf circumference. However, when comparing the baseline and 6-week follow-up, a significant decrease in calf circumference was observed in the control group. After 12 weeks, patients in the control group no longer showed a difference in calf circumference when compared to baseline. These changes of the calf as observed during the follow-up were according to the expected outcome, as long-term immobilization causes adaptive changes including atrophy and loss of strength, which are reversible after physical training [29, 30]. The risk of measurement bias were minimal as every calf circumference was constantly measured by the same investigator.

At the time of the initiation of this study, the weightbearing radiograph was not standard practice in [location blinded]. Ankle joint stability was assessed in a non-weightbearing radiograph by measuring medial clear space and was deemed unstable if this was > 6 mm. With the intervention group mobilizing within 10 days after fracture, there was need for elimination of possible false positive stable fractures because of the possibility of dislocation after weightbearing. In this study, assessment of the stability of the ankle joint was performed through physical and radiological examination. Notably, a total of 23 patients (comprising 16% of the total screened patients) who had been deemed eligible for conservative treatment based on the non-weightbearing mortise assessment were subsequently excluded due to the detection of medial clear space widening on the weightbearing Mortise assessment. Images depicting the ankle of a patient were added as supplementary material. These images demonstrate the transition from a stable ankle joint, as observed in non-weightbearing radiographs, to an unstable joint, as evident in weightbearing radiographs.

This study explores different aspects of fracture care in a randomized design, not only does it investigate ankle functionality after fracture, but it also aimed to explore quality of life differences between the intervention and control group. By using standardized outcome measures, we aimed to reduce the risk for detection bias.

Unfortunately, the study encountered difficulties with regard to patient inclusion, as several patients declined to participate due to various reasons. These reasons included apprehension about mobilization and weightbearing, concerns about joint instability during mobilization, and general fears of pain. Furthermore, some patients who were deemed fit for inclusion by the study's supervising surgeon were ultimately excluded due to pre-existing immobility. Another limitation pertains to the measurement of ankle functionality after 6 weeks: while patients in both the control and intervention group were asked to complete the OMAS approximately 30 min after removal of the cast or walker, patients in the control group were immobilized for 6 weeks and those in the intervention group were allowed to remove the walker from time to time at home and engage in physical exercise. Although a direct comparison between the two groups may not be appropriate at this time as the intervention group has been mobilizing for 6 weeks, while the control group has not had this opportunity yet, the study was designed to assess the differences in outcomes after 6 weeks. Cast-related stiffness is a well-known consequence of immobilization as previous research has shown that functional recovery after casting may take up to 10 weeks after immobilization [29]. The results of this study show that the OMAS of the control group remained comparable to the intervention group's score after 6 weeks, even after a 12-week follow-up period. This suggests that the difference in outcomes between the two groups would have likely persisted even if the patients in the control group had filled out the OMAS questionnaire 24 h later.

## Conclusion

Permissive weightbearing using a walking boot seems a safe and viable treatment for patients with stable Weber B fractures with regard to ankle functionality. This study highlights the potential advantages of using a walking boot such as increased patient satisfaction, improved self-care, and faster return to work.

### Supplementary Information

Below is the link to the electronic supplementary material.

Supplementary file1 (DOCX 1835 kb)

Supplementary file2 (DOCX 171 kb)

## Data Availability

Available upon request.
