# Supplementary material for: Prospective randomized controlled trial: early weight bearing after conservative treatment of Weber B ankle fractures (pancake trial)
Source: Eur J Orthop Surg Traumatol. 2023 Sep 2;34(1):591–8. doi: 10.1007/s00590-023-03651-6 (PMC10771350; doi:10.1007/s00590-023-03651-6)
Supplement: Supplementary file 1 — Supplementary file1 (DOCX 1835 kb) [file 590_2023_3651_MOESM1_ESM.docx]

**Supplement**

Image 1a: non-weightbearing lateral view 1 day after trauma.
Image 1b: non-weightbearing mortise view 1 day after trauma. Medial clear space is not widened.

Image 1c**:** non-weightbearing lateral view in cast, 1 day after trauma
Image 1d**:** non-weightbearing Mortise view in cast, 1 day after trauma


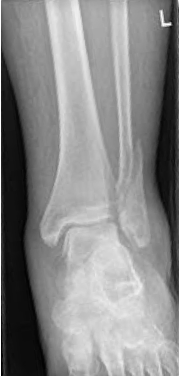

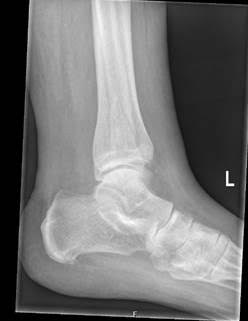


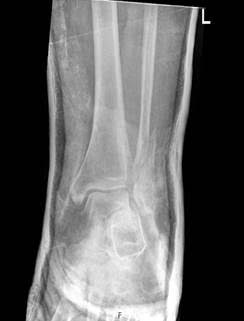

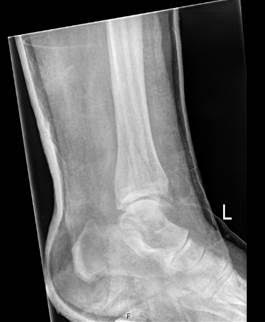


1d

1c

1b

1a

Image 2a: weightbearing lateral view one week post-trauma and after removal of cast


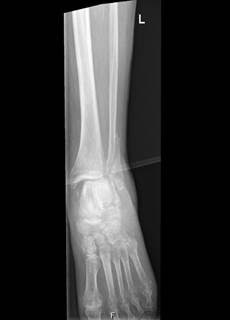

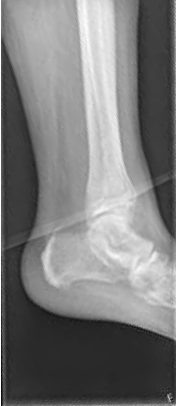
Image 2b:weightbearing mortise view one week post-trauma and after removal of cast

2a

2b
