# Supplementary material for: Prospective randomized controlled trial: early weight bearing after conservative treatment of Weber B ankle fractures (pancake trial)
Source: Eur J Orthop Surg Traumatol. 2023 Sep 2;34(1):591–8. doi: 10.1007/s00590-023-03651-6 (PMC10771350; doi:10.1007/s00590-023-03651-6)
Supplement: Supplementary file 2 — Supplementary file2 (DOCX 171 kb) [file 590_2023_3651_MOESM2_ESM.docx]

**PROSPECTIVE RANDOMIZED CONTROLLED TRIAL:**

**EARLY WEIGHT BEARING AFTER CONSERVATIVE TREATMENT OF WEBER B ANKLE FRACTURES (PANCAKE-TRIAL)**

| **Protocol ID** | **17-T-161** |
| --- | --- |
| **Short title** | **PANCAKE-trial** |
| **EudraCT number** | **Not applicable** |
| **Version** | **6** |
| **Date** | **06-06-2019** |
| **Coordinating investigator/project leader** | **Not applicable** |
| **Principal investigator** | **Dr. R. van Vugt** |
|  |  |
| **Sponsor** | **Zuyderland Medisch Centrum** |
|  |  |
| **Subsidising party** | **Not applicable** |
| **Independent expert** | **Dr. E.G. Boerma**  **Email address: e.boerma@zuyderland.nl** |
|  |  |
|  |  |
| **Laboratory sites** | **Not applicable** |
|  |  |
| **Pharmacy** | **Not applicable** |
|  |  |

**PROTOCOL SIGNATURE SHEET**

| **Name** | **Signature** | **Date** |
| --- | --- | --- |
| **Head of Department:**  ***Dr. L. Bouwman, Surgeon*** |  |  |
| **Principal Investigator:**  **Dr. R. van Vugt, Surgeon** |  |  |

**TABLE OF CONTENTS**

1. INTRODUCTION AND RATIONALE 7

2. OBJECTIVES 9

3. STUDY DESIGN 10

4. STUDY POPULATION 13

4.1 Population (base) 13

4.2 Inclusion criteria 13

4.3 Exclusion criteria 13

4.4 Sample size calculation 13

5. TREATMENT OF SUBJECTS 14

5.1 Investigational product/treatment 14

5.2 Use of co-intervention (if applicable) 14

5.3 Escape medication (if applicable) 14

6. INVESTIGATIONAL PRODUCT 15

7. NON-INVESTIGATIONAL PRODUCT 16

8. METHODS 17

8.1 Study parameters/endpoints 17

8.1.1 Main study parameter/endpoint 17

8.1.2 Secondary study parameters/endpoints (if applicable) 17

8.1.3 Other study parameters (if applicable) 17

8.2 Randomisation, blinding and treatment allocation 17

8.3 Study procedures 17

8.4 Withdrawal of individual subjects 18

8.4.1 Specific criteria for withdrawal (if applicable) 18

8.5 Replacement of individual subjects after withdrawal 18

8.6 Follow-up of subjects withdrawn from treatment 18

8.7 Premature termination of the study 18

9. SAFETY REPORTING 19

9.1 Temporary halt for reasons of subject safety………………..…………………………19

9.2 AEs, SAEs and SUSARs 19

9.2.1 Adverse events (AEs) 19

9.2.2 Serious adverse events (SAEs) 19

9.2.3 Suspected unexpected serious adverse reactions (SUSARs) 20

9.3 Annual safety report 20

9.4 Follow-up of adverse events 20

9.5 [Data Safety Monitoring Board (DSMB) / Safety Committee] 20

10. STATISTICAL ANALYSIS 21

10.1 Primary study parameter(s) 21

10.2 Secondary study parameter(s) 21

10.3 Other study parameters 21

10.4 Interim analysis (if applicable) 22

11. ETHICAL CONSIDERATIONS 23

11.1 Regulation statement 23

11.2 Recruitment and consent 23

11.3 Objection by minors or incapacitated subjects (if applicable) 23

11.4 Benefits and risks assessment, group relatedness 23

11.5 Compensation for injury 23

11.6 Incentives (if applicable) 23

12. ADMINISTRATIVE ASPECTS, MONITORING AND PUBLICATION 24

12.1 Handling and storage of data and documents 24

12.2 Monitoring and Quality Assurance 24

12.3 Amendments 24

12.4 Annual progress report 24

12.5 End of study report 24

12.6 Public disclosure and publication policy 25

13. STRUCTURED RISK ANALYSIS 26

14. REFERENCES 27

**LIST OF ABBREVIATIONS AND RELEVANT DEFINITIONS**

| **ABR** | **ABR form, General Assessment and Registration form, is the application form that is required for submission to the accredited Ethics Committee** |
| --- | --- |
| **AE** | **Adverse Event** |
| **ANCOVA** | **Analysis of Covariance** |
| **AP** | **Anteroposterior** |
| **AR** | **Adverse Reaction** |
| **ATTL** | **Anterior Talo-Tibial Ligament** |
| **CCMO** | **Central Committee on Research Involving Human Subjects; in Dutch: Centrale Commissie Mensgebonden Onderzoek** |
| **CT** | **Computed Tomography** |
| **CTCM** | **Clinical Trial Center Maastricht** |
| **CV** | **Curriculum Vitae** |
| **DSMB** | **Data Safety Monitoring Board** |
| **GCP** | **Good Clinical Practice** |
| **IC** | **Informed Consent** |
| **METC** | **Medical research ethics committee (MREC); in Dutch: medisch ethische toetsing commissie (METC)** |
| **PTTL** | **Posterior Talo-Tibial Ligament** |
| **ROM** | **Range of Motion** |
| **(S)AE** | **(Serious) Adverse Event** |
| **SE** | **Supination-Eversion** |
| **Sponsor** | **The sponsor is the party that commissions the organisation or performance of the research, for example a pharmaceutical company, academic hospital, scientific organisation or investigator.** |
| **SUSAR** | **Suspected Unexpected Serious Adverse Reaction** |
| **T1** | **First visit at the Zuyderland Medical Center** |
| **T2** | **Second visit at the Zuyderland Medical Center** |
| **T3** | **Third visit at the Zuyderland Medical Center** |
| **Wbp** | **Personal Data Protection Act (in Dutch: Wet Bescherming Persoonsgegevens)** |
| **WMO** | **Medical Research Involving Human Subjects** |
| **X^2^ Test** | **Chi-Squared Test** |

**SUMMARY**

**Rationale:** There is controversy about the optimal conservative treatment of common stable ankle fractures (Weber B or Lauge-Hansen supination-eversion stage 2-4A). The current international guideline describes non-weight bearing and immobilization with a plaster cast for 6 weeks. However, the literature provides no substantiation for this period of non-weight bearing.

Hypothesis 1: We expect earlier functional outcomes in the intervention group than in the control group. Hypothesis 2: We expect the number of complications not to be higher in the intervention group than in the control group. Hypothesis 3: We expect the long-term recovery function (3 months) in the intervention group not to be worse than in the control group. Null hypothesis 1: The treatment in the intervention group is not as effective as the treatment in the control group, because there are worse functional outcomes in the intervention group than in the control group. Null hypothesis 2: The treatment in the intervention group is not as safe as the treatment in the control group, because there are more dislocations of the fractures reported in the intervention group than in the control group.

**Objective**: What are the 6-week and 12-week effects on the functional outcome scores of the ankle in early weight bearing (mobilization with Walker) compared to 6 weeks non-weight bearing and immobilization in conservatively treated stable ankle fractures (Weber B or Lauge Hansen supination-eversion stage 2-4A)?

**Study design:** A prospective randomized controlled trial at the Zuyderland Medical Center . We will include patients at both locations, Heerlen and Sittard/Geleen.

**Study population:** Men and women, 16 years or older, with Weber B ankle fractures treated with a dorsal lower limb cast at the emergency department of both locations of the Zuyderland Medical Center, Heerlen and Sittard/Geleen, The Netherlands.

**Intervention:** One group will get a Walker by which they can start with permissive weight bearing. The other group will get a circular lower limb plaster cast and instructions of non-weight bearing and immobilization till the next visit. After 6 weeks, the Walker or plaster cast will be removed. The patients may then extend the weight bearing to functional.

**Main study parameters/endpoints:** The primary parameter in this study is functional outcome score of the ankle. Secondary parameter is dislocation of the fracture. Other parameters are range of motion of the ankle, circumference of the calf, return to work, satisfaction of the patient and other complications, such as deep vein thrombosis or surgery.

**Nature and extent of the burden and risks associated with participation***,* **benefit an group relatedness:** 3 site visits during 12 weeks with physical examinations and 2 questionnaires which have to be filled in during each visit. Risks of the investigational treatment are myalgia and dislocation of the fracture.

# INTRODUCTION AND RATIONALE

Ankle fractures are among the most common fractures of the lower extremities. With 9% of all fractures, they account for an important part of the traumatic injuries.^1^ A consideration between an operative vs. conservative treatment has to be made. A distinction must be made between stable and unstable ankle fractures.^2^ The fracture is stable if there is a dislocation of less than 3 millimetres at the ‘medial clear space’ (the jointspace between the medial malleolus and the talus) with a symmetrical ankle fork on the Mortise X-ray.

Classifications are used to describe the mechanism behind the ankle fracture and thus to make a statement about the (in)stability of the ankle joint. A common type of injury is the Weber B fracture or according to the Lauge-Hansen classification the supination-eversion type, that can be stable (SE type 2 without rupture of the deltoid ligament) and also possibly unstable (SE type 4 with rupture of the deltoid ligament). This ligament has a superficial and a deep layer. The superficial layer resists plantar flexion and external rotation of the talus relative to the tibia. The deep layer has anterior and posterior components, the anterior and posterior talo-tibial ligaments (ATTL and PTTL), of which the posterior ligament is the strongest. The PTTL is tight when the foot is plantigrade and loose when the foot is plantar flexed. If however, the foot is plantigrade, as on an anteroposterior weight-bearing radiograph, the intact PTTL will prevent lateral translation of the talus and the ankle fork appears congruent.^3^ Therefore, Gougoulias N. et al. (2017) proposed that a (ligamentous) SE type 4 ankle fracture, one without a medial malleolar fracture, with a ruptured superficial and/or ATTL, but an intact PTTL, can be classified as a ‘SE type 4A’ fracture. This is a stable fracture, as long as the foot is in a plantigrade position. With a complete rupture of the superficial and deep components (both ATTL and PTTL) of the deltoid ligament, the medial clear space will open in all positions of the foot, thus also on weight-bearing radiographs. This type of fracture can be classified as SE type 4B, which is always an unstable fracture.

There is controversy about the optimal conservative treatment of common stable ankle fractures. This is confirmed by the presence of a large number of treatment protocols with great variability in weight bearing recommendations.^4^ The current international guideline describes non-weight bearing and immobilization with a plaster cast for 6 weeks.^5^ However, a prolonged period of immobilization causes various negative effects regarding recovery:

- *Patient compliance*: The noncompliance rate with the postoperative weight bearing restriction is almost 30% and they start with weight bearing despite explicit instructions. However, this rarely leads to displacement of the fracture. The advice currently being applied may therefore be too cautious.

# *Physiological cost*: Non-weight bearing with 2 elbow crutches and only 1 leg on the ground costs 4 times more energy than walking with 2 legs on the ground and 2 crutches for support or stabilization.

# *Homeostasis:* Weight bearing ensures the preservation of bone and muscle mass. A few weeks of non-weight bearing results in a significant decrease in bone mass in the affected extremity even a year later.^6^

# *Risk of thrombosis:* Posttraumatic immobilization with a plaster cast is a risk factor for developing a deep venous thrombosis or a pulmonary embolism. At Zuyderland Medical Center, patients receive thromboprophylaxis during long term immobilization, according to protocol.

# Therefore, advising non-weight bearing out of caution or uncertainty can cause harmful effects on the health of patients. However, the literature provides no substantiation for this period of non-weightbearing.^7^ When transmitting anxiety to patients, this can make them insecure, which does not contribute to rehabilitation.

# Previous findings: According to our knowledge, there has not been previously performed a prospective randomized comparative study on early weight bearing in conservatively treated stable ankle fractures (Weber B of Lauge Hansen SE2-4A). Van Laarhoven^8^ refers in his thesis to two studies^9,10^ in which early weight bearing of stable Weber B fractures yields good to excellent functional results. However, both studies have a limited size of the study population and control groups are lacking.

# Relevance: According to an epidemiological study of Court-Brown et. Al, Weber B ankle fractures (Lauge Hansen SE2) with 21% have the highest incidence within the total number of ankle fractures (n=1500).^11^ According to the current trend, more and more rehabilitation programs use early weight bearing. However, this does not apply to this large group of patients if the current protocols continue to be used. If our hypotheses are correct, old recommendations about long term immobilization could be abandoned and we can opt for a more patient-oriented follow-up treatment.

# OBJECTIVES

**Objective:** What are the 6-week and 12-week effects on the functional outcome scores of the ankle in early weight bearing (mobilization with Walker) compared to 6 weeks non-weight bearing and immobilization in conservatively treated stable ankle fractures (Weber B or Lauge Hansen supination-eversion stage 2-4A)?

**Secondary Objective:** What is the 6-week effect on dislocation of the fracture in early weight bearing (mobilization with Walker) compared to 6 weeks non-weight bearing and immobilization in conservatively treated stable ankle fractures (Weber B or Lauge Hansen supination-eversion stage 2-4A)?

**Other Objectives:** Other outcome measures include range of motion (ROM) of the ankle circumference of the calf, return to work, mental health outcome scores and rates of other complications.

**Hypothesis 1:** We expect earlier functional outcomes in the intervention group than in the control group.

**Hypothesis 2:** We expect the number of complications not to be higher in the intervention group than in the control group.

**Hypothesis 3:** We expect the long-term recovery function (3 months) in the intervention group not to be worse than in the control group.

**Null hypothesis 1**: The treatment in the intervention group is not as effective as the treatment in the control group, because there are worse functional outcomes in the intervention group than in the control group.

**Null hypothesis 2:** The treatment in the intervention group is not as safe as the treatment in the control group, because there are more dislocations of the fractures reported in the intervention group than in the control group.

# STUDY DESIGN

A prospective randomized controlled trial at the Zuyderland Medical Center. We will include patients at both locations, Heerlen and Sittard/Geleen.

All patients with ankle fractures at the emergency department of both locations of the Zuyderland Medical Center are treated with a dorsal lower limb cast. 7 to 10 days posttraumatic, they are seen again at the traumatology department, according to protocol.

During this visit ***(= t1)****,* radiographs of the ankle joint are obtained at 3 different views (anteroposterior (AP), lateral and Mortise) and a weight-bearing radiograph (AP). When it’s a stable fracture according to the surgeon, present at that time, the patients will be approached to participate in this randomized study. If written informed consent is obtained, the patients get included.

**Intervention group**

The dorsal lower limb cast will be removed by the plaster technician. Patients will get a Walker by which they can start with permissive weight bearing. According to protocol, they receive daily thromboprophylaxis (Fragmin 5000IE s.c.). After 6 weeks posttraumatic, the Walker will be removed at the traumatology department. The patients may then extend the weight bearing to functional.

**Control group**

The dorsal lower limb cast will be removed by the plaster technician and a circular lower limb plaster cast will be applied. Patients get explicit instruction of non-weight bearing and immobilization till the next visit. According to protocol, they receive daily thromboprophylaxis (Fragmin 5000IE s.c.). After 6 weeks posttraumatic, the plaster cast will be removed at the traumatology department. The patients may then start weight bearing to their own abilities.

**Follow-up**

The patients will visit the traumatology department after 6 weeks ***(= t2)*** and 12 weeks ***(= t3)*** posttraumatic. During the visit after 6 weeks, radiographs of the ankle joint are obtained at 3 different views (anteroposterior (AP), lateral and Mortise). Furthermore, they will undergo a comprehensive examination that will include assessment of ankle dorsal flexion/ plantar flexion with use of a goniometer. The circumference of the calf will be measured with a tapeline. This will be performed by the trained research staff or by doctors trained and instructed by them.

Each visit ***(t1, t2 ánd t3)*** the patients receive 2 questionnaires with regard to return to work or sport, daily activities and limiting factors.


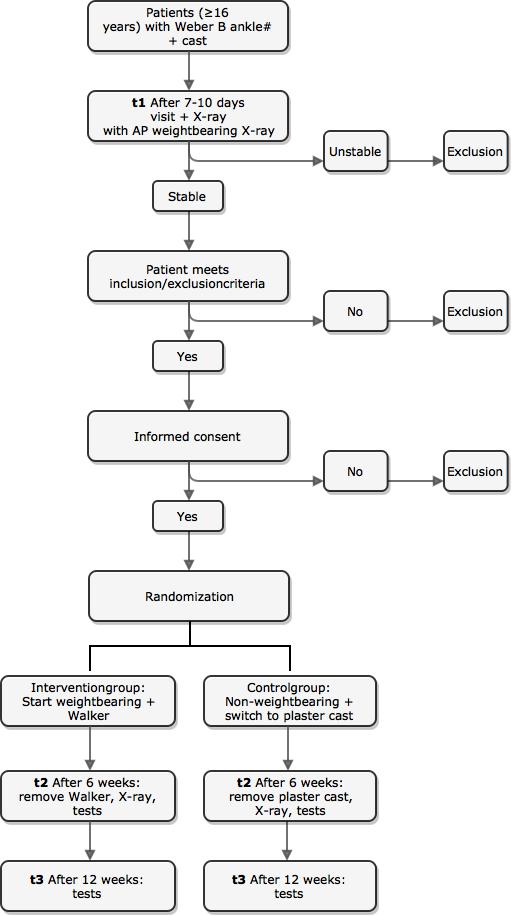


Figure 1. Flowchart


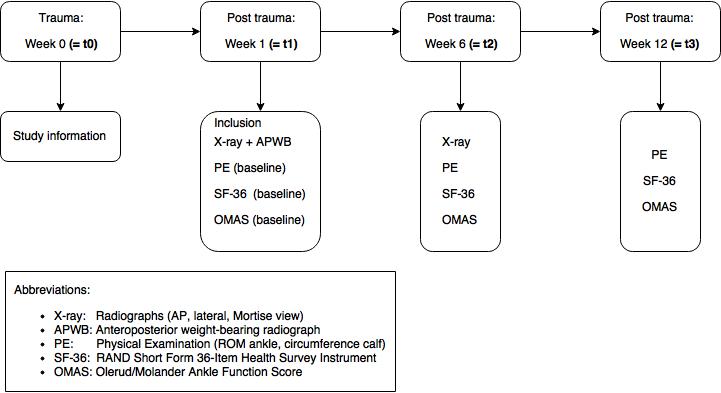


Figure 2. Timeline

| Variable (outcome measure) | Type | t1 | t2 | t3 | Total |
| --- | --- | --- | --- | --- | --- |
| Functional outcome score (OMAS, 0-100) | continuous | ✓ | ✓ | ✓ | 3 |
| Dislocation of the fracture (Yes/No) | binary | ✓ | ✓ | × | 2 |
| Range of motion of the ankle (degrees) | continuous | ✓ | ✓ | ✓ | 3 |
| Circumference of the calf (cm) | continuous | ✓ | ✓ | ✓ | 3 |
| Return to work (%) | continuous | ✓ | ✓ | ✓ | 3 |
| Satisfaction (SF-36, 0-100) | continuous | ✓ | ✓ | ✓ | 3 |
| Other complications | nominal | × | ✓ | ✓ | 2 |

Table 1. Measurements

# STUDY POPULATION

## Population (base) Men and women with a Weber B or isolated malleolus tertius ankle fracture treated with a dorsal lower limb cast at the emergency department of both locations of the Zuyderland Medical Center, Heerlen and Sittard/Geleen, The Netherlands.

## Inclusion criteria

In order to be eligible to participate in this study, a subject must meet all of the following criteria:

- Age of 16 years and older.
- Stable ankle fracture (non-dislocated Weber B or Lauge Hansen supination-eversion stage 2-4A fracture, isolated malleolus tertius fracture).
- Conservative treatment with a dorsal lower limb cast.

## Exclusion criteria

A potential subject who meets any of the following criteria will be excluded from participation in this study:

- Age below 16 years.
- Weber A ankle fracture.
- Unstable ankle fracture.
- Fractures involving both lower extremities.
- Operative treatment of the ankle fracture.
- Posttraumatic period more than 14 days.
- Amputation of upper leg, lower leg or foot.

## Sample size calculation

The primary outcome measure is the Olerud/Molander Ankle Function Score which is continuous variable. A recent study^12^ compared the same intervention on post-operative ankle fractures using the same outcome measure. We used those means and standard deviations to determine our sample size. Using the ‘EpiTools epidemiological calculator’ it was calculated that 23 patients per group would be needed to obtain 80% power with an P-value of 0.05. Accounting for a 10% drop out rate, we are planning to recruit 46 patients per group, for a total of 50 patients in the study.

# TREATMENT OF SUBJECTS

## Investigational product/treatment

##
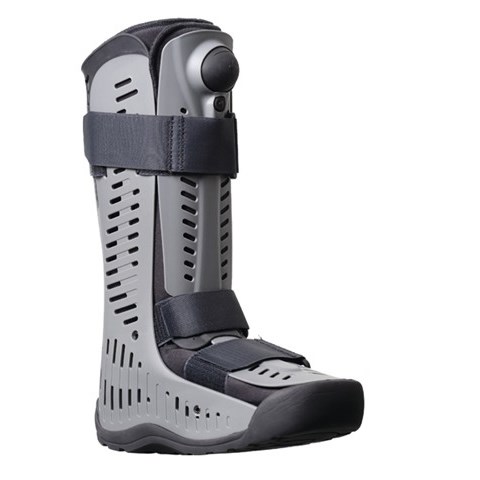
The Rebound®Air Walker is light and strong with a high degree of comfort and stability. The foot is kept in a stable plantigrade position. The advantages of a Rebound® Air Walker, above a plaster cast, are the possibilities of showering, intermediate exercise and a decrease in oedema of the ankle joint due to the built-in air compression system. Integrated pumps with coupled air cushions on the inside and outside sides ensure even compression. A valve lets the air escape when the Air Walker is removed.

Figure 3. Rebound® Air Walker

## Use of co-intervention (if applicable)

Not applicable.

## Escape medication (if applicable)

Not applicable.

# INVESTIGATIONAL PRODUCT

Not applicable.

# NON-INVESTIGATIONAL PRODUCT

Not applicable.

# METHODS

## Study parameters/endpoints

### Main study parameter/endpoint

Functional outcome scores of the ankle. This will be indicated with the Olerud/Molander Ankle Function Score (OMAS). This is a continuous variable (0-100 points).

### Secondary study parameters/endpoints

Dislocation of the fracture within 6 weeks (after placement of the Walker or circular lower limb plaster cast). This is a dichotomous variable (yes/no).

### Other study parameters

Range of motion (ROM) of the ankle and circumference of the calf (physical examination), return to work, mental health outcome scores (SF-36 questionnaire) and other complications, such as deep vein thrombosis or surgery.

## Randomisation, blinding and treatment allocation

Patients are randomized with baseline stratification (by gender, age, smoking status and diabetes) into the intervention group or the control group. For concealed allocation of the participants, a computer-generated list of random numbers will be used, which is accessible at both Zuyderland locations. Blinding of patients and researchers is not possible in this case.

## Study procedures

| Procedures | Types |
| --- | --- |
| Radiographs | Anteroposterior(AP), lateral and Mortise view |
|  | Weight-bearing radiograph(AP) |
| Questionnaires | Olerud/Molander Ankle Function Score* |
|  | RAND Short Form 36-Item Health Survey Instrument* |
| Physical examination | Ankle dorsal flexion/ plantar flexion, with use of a goniometer. |
|  | The circumference of the calf, with use of a tapeline.***** |

********Extra procedures for this study.*

## Withdrawal of individual subjects

Subjects can leave the study at any time for any reason if they wish to do so without any consequences. The investigator can decide to withdraw a subject from the study for urgent medical reasons.

### Specific criteria for withdrawal (if applicable)

Not applicable.

## Replacement of individual subjects after withdrawal

## Not applicable.

## Follow-up of subjects withdrawn from treatment

## All data obtained until the moment of withdrawal will be taken into account in the data analysis.

## Premature termination of the study

## We don’t expect any (adverse) events that will result in premature termination of the study.

# SAFETY REPORTING

## Temporary halt for reasons of subject safety

In accordance to section 10, subsection 4, of the WMO, the sponsor will suspend the study if there is sufficient ground that continuation of the study will jeopardise subject health or safety. The sponsor will notify the accredited METC without undue delay of a temporary halt including the reason for such an action. The study will be suspended pending a further positive decision by the accredited METC. The investigator will take care that all subjects are kept informed.

## AEs, SAEs and SUSARs

### Adverse events (AEs)

Adverse events are defined as any undesirable experience occurring to a subject during the study, whether or not considered related to the experimental intervention]. All adverse events reported spontaneously by the subject or observed by the investiga­tor or his staff will be recorded.

### Serious adverse events (SAEs)

A serious adverse event is any untoward medical occurrence or effect that

- results in death;
- is life threatening (at the time of the event);
- requires hospitalisation;
- results in persistent or significant disability or incapacity;
- any other important medical event that did not result in any of the outcomes listed above due to medical or surgical intervention but could have been based upon appropriate judgement by the investigator.

An elective hospital admission will not be considered as a serious adverse event.

The sponsor will report the SAEs through the web portal *ToetsingOnline* to the accredited METC that approved the protocol, within 7 days of first knowledge for SAEs that result in death or are life threatening followed by a period of maximum of 8 days to complete the initial preliminary report. All other SAEs will be reported within a period of maximum 15 days after the sponsor has first knowledge of the serious adverse events.

### Suspected unexpected serious adverse reactions (SUSARs)

### Not applicable.

## Annual safety report

## Not applicable.

## Follow-up of adverse events

All AEs will be followed until they have abated, or until a stable situation has been reached. Depending on the event, follow up may require additional tests or medical procedures as indicated, and/or referral to the general physician or a medical specialist.

SAEs need to be reported till end of study within the Netherlands, as defined in the protocol

## Data Safety Monitoring Board (DSMB)

The Clinical Trial Center Maastricht (CTCM) will be monitoring the study for its safety. It’s an independent committee and it has no conflict of interest with the sponsor of the study . The advice(s) of the DSMB will only be sent to the sponsor of the study. Should the sponsor decide not to fully implement the advice of the DSMB, the sponsor will send the advice to the reviewing METC, including a note to substantiate why (part of) the advice of the DSMB will not be followed.

# STATISTICAL ANALYSIS

The baseline values (gender, age, quality of life, smoking status, diabetes) will be analysed and explained in table 1 of the article. The means and standard deviations of the continuous variables and the numbers and percentages of the categorical variables will be measured. A P-value will be indicated by the use of an unpaired t-test (continuous variables) and a X^2^ test (categorical variables). Variables at baseline that show (statistical significant) differences between intervention group and control group, will be added in the multivariate analysis.

## Primary study parameter(s)

The continuous variables will be presented as quantitative variables. The ANCOVA will be used to adjust for baseline values. A P-value of less than 0.05 was considered statistically significant. Patients were analysed based on per protocol principles.

## Secondary study parameter(s)

The categorical data will be presented as qualitative variables. Starting with a univariate analysis, the X^2^ test will be used for the binary variables. A risk ratio, including 95% confidence intervals, will be used to indicate the probability of a dislocation of the fracture in comparison to both groups. Thereafter, we will use a multivariate logistic regression analysis, at which the odds ratio will adjust for baseline values. A P-value of less than 0.05 was considered statistically significant. Patients were analyzed based on per protocol principles.

If there is a suspicion of a dislocation of the fracture, based on the assessment of the radiograph and low functional outcome scores, a Computed Tomography (CT) of the ankle will be made to confirm this. Thus, we want the type I error to be controlled.

## Other study parameters

The continuous variables will be presented as quantitative variables. The ANCOVA will be used to adjust for baseline values. The X^2^ test will be used for nominal variables. A P-value of less than 0.05 was considered statistically significant. Patients were analysed based on per protocol principles.

## Interim analysis (if applicable)

At the end of April 2018 an interim analysis will be done by the investigator. The same statistical methods will be used as described in Chapter 10.1, 10.2 and 10.3. The advice(s) of the DSMB will be taken into account and implemented if necessary.

# ETHICAL CONSIDERATIONS

## Regulation statement

The study will be conducted according to the principles of the Declaration of Helsinki (64^th^ WMA General Assembly, October 2013) and in accordance with the Medical Research Involving Human Subjects Act (WMO).

## Recruitment and consent

All patients with ankle fractures at the emergency department of both locations of the Zuyderland Medical Center will receive information for participants on sight. The investigator informs the emergency doctors in advance to make a note in the patient files to confirm that the patients are informed about this study. Once a week the investigator will check if the patients got informed about the study. If not, the investigator will call the patients and he will send the participant information form by e-mail.

During their next visit after 7 to 10 days the patients are asked for their consent (see appendix).

## Objection by minors or incapacitated subjects (if applicable)

Not applicable.

## Benefits and risks assessment, group relatedness

Benefits of the investigational treatment can be an earlier return to work or sport. Risks of the investigational treatment are myalgia and dislocation of the fracture. We expect the the latter to occur rarely, if ever.

## Compensation for injury

The sponsor/investigator has a liability insurance which is in accordance with article 7 of the WMO.

The sponsor (also) has an insurance which is in accordance with the legal requirements in the Netherlands (Article 7 WMO). This insurance provides cover for damage to research subjects through injury or death caused by the study.

The insurance applies to the damage that becomes apparent during the study or within 4 years after the end of the study.

## Incentives (if applicable)

Not applicable.

# ADMINISTRATIVE ASPECTS, MONITORING AND PUBLICATION

## Handling and storage of data and documents

Data will be handled confidentially and anonymously. A subject identification code list will be used to link the data to the subject. The code is not based on the patient initials and birth-date. The key to the code will be safeguarded by the investigator.

## Monitoring and Quality Assurance

The Clinical Trial Center Maastricht (CTCM) will be monitoring the study.

## Amendments

Not applicable.

## Annual progress report

The sponsor/investigator will submit a summary of the progress of the trial to the accredited METC once a year. Information will be provided on the date of inclusion of the first subject, numbers of subjects included and numbers of subjects that have completed the trial, serious adverse events/ serious adverse reactions, other problems, and amendments.

## Temporary halt and (prematurely) end of study report

The investigator will notify the accredited METC of the end of the study within a period of 8 weeks. The end of the study is defined as the last patient’s last visit.

The sponsor will notify the METC immediately of a temporary halt of the study, including the reason of such an action.

In case the study is ended prematurely, the sponsor will notify the accredited METC within 15 days, including the reasons for the premature termination.

Within one year after the end of the study, the investigator/sponsor will submit a final study report with the results of the study, including any publications/abstracts of the study, to the accredited METC.

## Public disclosure and publication policy

We will publish the results with a national and international article. If the inclusion is not achieved within the stipulated time, we will make a report of the preliminary results. We hope to be able to present the results of this research at regional or national level.

# STRUCTURED RISK ANALYSIS

Not applicable.

# REFERENCES

1. Singh R, Kamal T, Roulohamin N, Maoharan G, Ahmed B, Theobald P; Ankle Fractures: A Literature Review of Current Treatment Methods. Open Journal of Orthopedics, 2014, 4, 292-303

2. Michelson JD, Magid D, McHale K. Clinical utility of a stability-based ankle fracture classification system. J Orthop Trauma. 2007 May;21(5):307-15.

3. Gougoulias N. When is a simple fracture of the lateral malleolus not so simple? Bone Joint J 2017;99-B:851–5.

4. Pfeifer CG, Grechenig S, Frankewycz B, Ernstberger A, Nerlich M, Krutsch W. Analysis of 213 currently used rehabilitation protocols in foot and ankle fractures. Injury. 2015 Oct;46 Suppl 4:S51-7. doi: 10.1016/S0020-1383(15)30018-8.

5. Rüedi TP, Buckley RE, Moran CG. AO principles of fracture management. 2e uitgebreide dr. New York: Thieme; 2007.

6. Brink PRG., Verleisdonk EJMM., Blokhuis TJ. Eerder belast mobiliseren na fractuurfixatie; NTVG 2017;161: D1533

7. Haller JM, Potter MQ, Kubiak EN. Weight bearing after a periarticular fracture: what is the evidence? Orthop Clin North Am. 2013;44:509-19.

8. Laarhoven van CJHM. Fractures of the ankle joint. Retrospective and prospective studies on the (long-term) results of protocolled treatment. Dissertation. Utrecht, The Netherlands 1994.

9. Veldhuizen JW, van Thiel TP, Oostvogel HJ, Stapert JW. Early functional treatment of supination-eversion stage-II ankle fractures: preliminary results. Neth J Surg. 1988 Dec;40(6):155-7.

10. Zeegers AV, Van Raay JJ, van der Werken C. Ankle fractures treated with a stabilizing shoe. Acta Orfhop Scand 1989;60(5):597-9

11.Court-Brown CM, McBirnie J and Wilson G. Adult ankle fractures-an increasing problem? Acta Orfhop Scand 1998; 69 (1): 43-47

12. Dehghan N, McKee MD, Jenkinson RJ, Schemitsch EH, Stas V, Nauth A, Hall JA, Stephen DJ, Kreder HJ. Early weightbearing and range of motion versus non-weightbearing and immobilization after open reduction and internal fixation of unstable ankle fractures: A randomized controlled trial.
